# Root Microbiota: Orchestrating Architecture‐Smart Crops

**DOI:** 10.1111/1751-7915.70307

**Published:** 2026-02-03

**Authors:** Qinqin Chen, Yanlai Yao, Huan Chen, Baolei Jia

**Affiliations:** ^1^ Xianghu Laboratory Hangzhou China; ^2^ Educational Ministry Engineering Centre of Resource‐Saving Fertilisers Nanjing Agricultural University Nanjing China; ^3^ School of Agriculture and Biology Shanghai Jiao Tong University Shanghai China

**Keywords:** architecture, crop architecture, Cyclo(Leu‐Pro), root microbiota, strigolactone

## Abstract

Crops depend on microbial partners for their growth, development, and overall resilience. A pivotal understanding has emerged showing the direct involvement of the root microbiota in regulating the tiller number of rice, a crucial architecture that influences yield. Novel frontiers in microbiological applications for agriculture highlight the profound role of the root microbiota in shaping crop architecture to boost productivity. We propose that improvements in crop production are moving from a genetic perspective on “architecture” to embracing “holobiont architecture.” As such, microbial orchestration provides a dynamic fine‐tune function for breeding “architecture‐smart crops” characterised by phenotypic plasticity under environmental uncertainty.

## Graphical abstract

Root‐associated microbiota play an important role in shaping crop architecture. The concept of “holobiont architecture” is proposed, in which plant architecture is dynamically fine‐tuned through synergistic plant–microbiome interactions. This framework offers novel avenues for breeding architecture‐smart crops with improved phenotypic plasticity and environmental resilience.

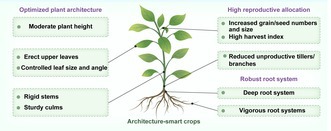

## 1 Introduction

The root microbiota affects the growth and development of plants and plays an important role in plant health and tolerance to environmental stress (Berruto and Demirer 2024; Compant et al. 2025). Root‐associated microbiota can induce resistance to pathogens through coordinated regulation of host immunity and rhizosphere community structure (Ge et al. 2024). Notably, the root microbiome is often referred to as the “second genome” of plants. The bulk of existing research has largely focused on the role of root microbiota in crop development and stress tolerance through facilitating nutrient acquisition, synthesising phytohormones, and enhancing root development (Liu et al. 2025). However, our understanding of how the root microbiota affects crop architecture and key yield components is limited. The root microbiota has recently been found to directly influence the number of rice tillers, which is a crucial aspect of crop architecture that significantly impacts yield (Zhang et al. 2025). This emerging evidence challenges the view of crop architecture as a static, genetically predetermined “architecture,” focusing on optimising yield and morphological traits. We suggest that the genetically defined crop architecture is a static framework and that its full adaptive potential can be realised through dynamic microbial regulation. We further propose a “holobiont architecture,” in which phenotypic plasticity and fine‐tuning can be realised through targeted regulation of the rhizosphere microbiome, thereby cultivating “architecture‐smart crops.” This opinion paper explores this hypothesis, provides evidence for microbial regulation, and proposes a research roadmap for leveraging microbe and plant architecture to advance sustainable agriculture.

## 2 Root Microbiota's Role in Crop Architecture

**FIGURE 1:**
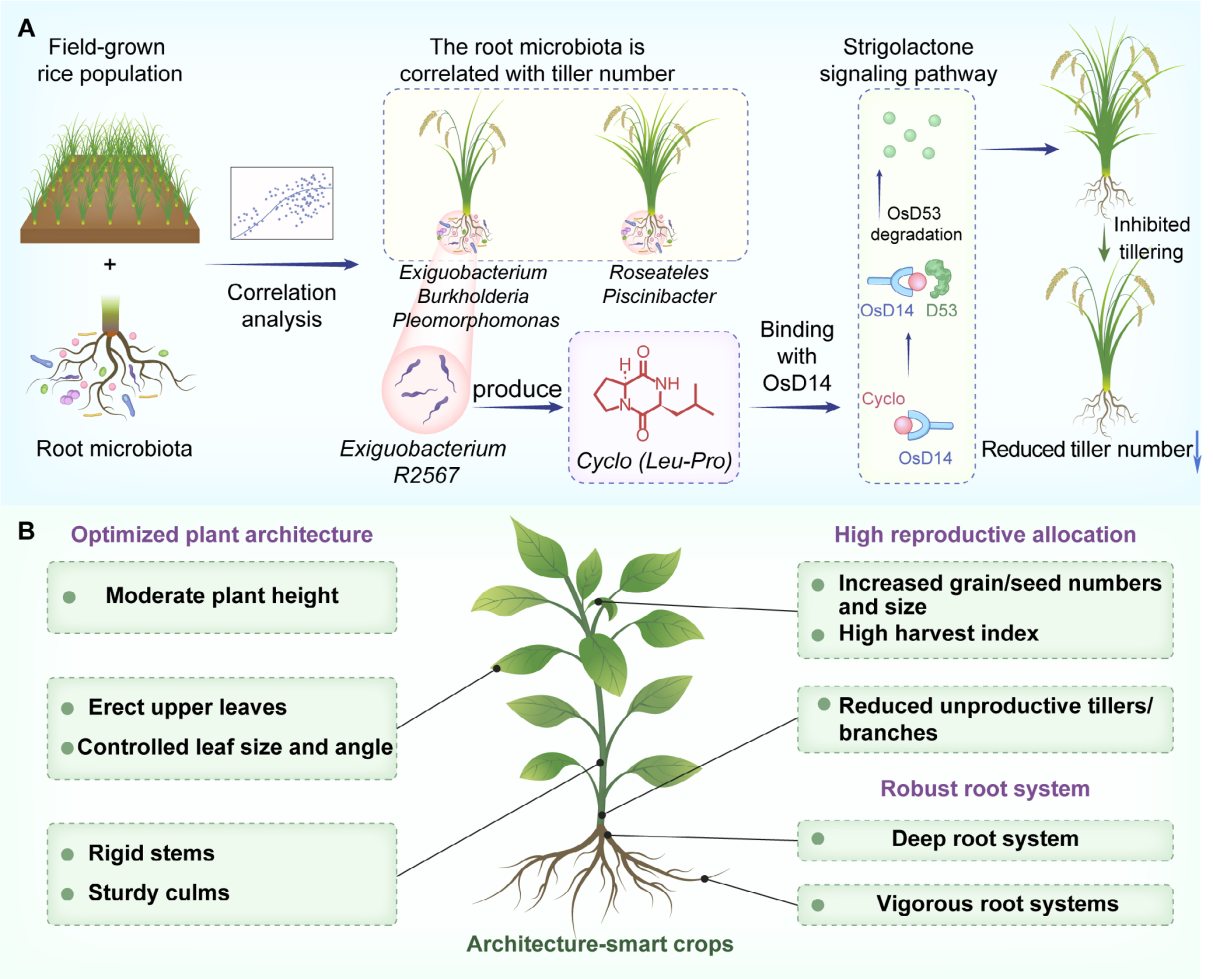
Contribution of the plant microbiota to architecture‐smart crops. (A) Root microbiota regulate rice tiller number. Analysis of associations between rice tiller number and root microbiota in a field‐grown population revealed that *Roseateles* R780 and *Piscinibacter* R1801 increased tiller number, while *Exiguobacterium* R2567 decreased it. *Exiguobacterium* R2567 produces Cyclo(Leu‐Pro), which regulates rice tillering by promoting the degradation of the strigolactone signalling repressor OsD53 via binding to D14 receptor complexes. (B) The plant microbiota's potential to create architecture‐smart crops. An architecture‐smart crop is characterised by optimised shoot architecture, high reproductive allocation, and a robust root system. The plant microbiota plays a vital role in contributing to these desirable traits, thereby fostering the development of architecture‐smart crops.

Crop architecture, encompassing traits such as plant height, leaf angle, and branching patterns (tillering in cereals), is a primary determinant of photosynthetic efficiency, resource allocation, and grain yield (Wang et al. 2018). In rice, tillering directly dictates the number of productive panicles and overall harvest. Although plant hormones, particularly strigolactones (SLs), have long been known to regulate shoot branching, Zhang et al. introduced a dynamic layer of external control exerted by the root microbiota (Figure 1A) (Zhang et al. 2025). Specifically, Zhang et al. performed a comprehensive analysis of field‐grown rice populations and revealed a correlation between the distinct composition of the root microbiota and variations in tiller number across diverse rice varieties. Additionally, they performed targeted functional assays through meticulous isolation and cultivation of individual bacterial strains from the rice root microbiota and identified 12 bacterial genera (seven positive, five negative) as key regulators in two independent field trials (Zhang et al. 2025). Functional validation showed that *Roseateles* R780 and *Piscinibacter* R1801 enhanced tillering, whereas *Exiguobacterium* R2567, *Burkholderia* R2488, and *Pleomorphomonas* R1405 suppressed it. Notably, the positive effect of *Roseateles* R780 depended on SL biosynthesis and signalling, whereas the inhibitory effect of *Exiguobacterium* R2567 on tillering primarily targeted SL signalling.

Further mechanistic investigations revealed that *Exiguobacterium* R2567 achieves its architectural modulation by producing a unique dipeptide molecule, Cyclo(Leu‐Pro). This small molecule was identified as the key effector responsible for activating the SL signalling pathway, which suppresses tiller formation. Cyclo(Leu‐Pro) activates the SL signalling pathway by directly binding to OsD14, the rice SL receptor. This precise molecular binding event triggers a downstream signalling cascade, leading to the degradation of the inhibitory protein OsD53. This process ultimately inhibits tillering, thereby effectively mimicking the physiological effects of endogenous SLs. Overall, these findings reveal how a bacterial metabolite precisely docks with and synergistically regulates the complex hormonal machinery of the host, thereby affecting the molecular mechanism of key developmental programs. This precise interaction at the molecular level highlights the previously underestimated level of exquisite regulation in plant‐microbe interactions.

Notably, the fine‐tuning of plant architecture by root microorganisms is not limited to the shoot systems, but it also extends to the subterranean structures. Recently, Fu et al. (2025) found that the bacillolysin, a protein secreted by *Bacillus velezensis* SQR9, can promote the growth of *Arabidopsis* lateral roots. Mechanistically, bacillolysin mediates the IAA34‐PUCHI module, in which IAA34 is a transcriptional repressor and PUCHI is a transcriptional factor, thereby activating downstream auxin response genes by interacting with the *Arabidopsis* receptor CEPR2 (Fu et al. 2025). Auxin, another fundamental plant hormone, is a well‐known regulator of root system architecture. However, lateral root regulation by microorganisms is not restricted to auxin‐dependent pathways. Resident microbiota can reprogram lateral root branching through an evolutionarily conserved, auxin‐independent mechanism that primarily engages ethylene signalling, revealing an additional layer of microbial control over belowground architectural plasticity that enhances plant resilience to environmental heterogeneity (Gonin et al. 2023).

Beyond these aspects of root system architecture, microbial regulation of plant architecture extends to additional developmental modules. *P. fluorescens* and *Root918* can fine‐tune the lamina: petiole ratio (leaf‐to‐stalk ratio) under shade, which facilitates the allocation of carbon resources to photosynthetic tissue rather than structural elongation (Lu et al. 2025). In maize, lateral‐root‐enriched Massilia (*Oxalobacteraceae*) acts as a keystone taxon linking root system differentiation with above‐ground traits such as flowering time and biomass accumulation, providing direct evidence that spatially structured root microbiota participate in shaping crop architectural plasticity (Wang et al. 2024).

Collectively, these findings highlight a pervasive and tightly regulated capacity of the plant microbiota to manipulate host plant morphology. Such microbial orchestrators hold immense potential for engineering specific plant traits through targeted interventions.

## 3 Ideal Architectype for Crops

Optimising plant architecture, or the ‘architectype’, is considered a cornerstone of the “next Green Revolution” (Li et al. 2025). Moreover, research on maize smart‐canopy architecture underscores the critical importance of optimising traits, such as leaf angle, for enhanced yield at high planting densities (Tian et al. 2024). This provides a clear target trait that could potentially be influenced through both traditional plant breeding and innovative microbial interventions, adding another layer of complexity and potential to crop improvement strategies. Rice, wheat, corn, and soybeans are different staple crops, but their ideal architectypes share common characteristics for improving yield and resource utilisation (Figure 1B). The ideal architectype for the optimised shoot system includes moderate tillering, reduced height, strengthened stems to prevent lodging, and efficient leaf display to maximise light energy capture. The ideal architectype also prioritises high reproductive allocation, focusing resources on increasing the number and size of grains or seeds while minimising unproductive growth, resulting in a high harvest index. Finally, the ideal architectype should have a strong and deep root system to ensure efficient nutrient and water absorption and enhance the overall resilience of the plant (Figure 1B). These characteristics collectively define the essential features of an “architecture‐smart crop” designed for optimal yield and resource efficiency.

## 4 From Static Architecture to Dynamic Holobiont Architecture

The “plant architecture” concept has conventionally focused on optimising genetically determined morphological traits, such as plant height, leaf angle, and branching patterns, to maximise yield under specific conditions. This framework has played a vital role in breeding high‐yielding wheat varieties and semi‐dwarf rice. Under this perspective, plant architecture has been recognised as a static outcome of the plant genome. However, this viewpoint overlooks the dynamic regulatory function provided by the microbiome (Kostic et al. 2024; Michan and Ramos 2025). Herein, we introduce the concept of “holobiont architecture,” where the final plant architecture is a product of its genetic elements and microbial patterners. The plant microbiome can be recognised as an external regulatory system that regulates key developmental and stress response pathways, actively shaping plant architecture to respond to environmental signals. This paradigm shifts our focus from a static genetic factor to a dynamic and responsive system, where microbial partners act as external regulators of plant architecture. By systematically elucidating the molecular mechanisms of this partnership and utilising tools such as synthetic microbial communities (SynComs) and hologenome breeding, scientists can develop “architecture smart crops.” These crops will be able to proactively adjust their architecture to optimise resource use, enhance stress resistance, and maintain high yields in the face of environmental uncertainty.

## 5 Microbiological Avenues for Architecture‐Smart Crops

To integrate microbiome with breeding, architecture smart crops could be optimised from three interrelated research perspectives: (1) deciphering the “chemical language” between microbes and plants; (2) designing functionally SynComs capable of predictively regulating plant architecture; and (3) including “microbiome responsiveness” as a key agronomic trait. By synergistically applying these methods, it would be possible to cultivate crops that can dynamically adjust their architecture based on microbial patterners, thus ultimately improving crop productivity and adaptability in variable agricultural environments.

We suggest that there is a conserved “chemical language”—a suite of microbial metabolites and proteins—that is used by diverse microbes to modulate plant architecture. The dipeptide cyclo(Leu‐Pro) and the bacillolysin are the first words that have been deciphered in this language. Future research could employ high‐throughput screening, metabolomics, and proteomics to systematically identify novel microbial‐derived molecules (volatile organic compounds, amino acid derivatives, hormone derivatives, peptides, secreted proteins, and other diverse small molecules) that regulate key architectural traits such as leaf angle, stem height, root depth, and branching patterns. Elucidating their biosynthesis pathways and identifying their specific plant receptors will be critical for future biotechnological applications.

Functionally, SynComs promise to predictively regulate crop architecture by moving from single‐strain inoculums to microbial “teams.” The designed SynComs require functional complementarity, where different members contribute unique and coordinated activities to modulate target traits. For example, a consortium containing a precise ratio of tiller‐promoting (e.g., *Roseateles*) and tiller‐inhibiting (e.g., *Exiguobacterium*) bacteria could be applied to fine‐tune panicle number in rice based on planting density and nutrient availability. Factually, the application of SynComs with *Azospirillum* and *Bacillus* could elevate tiller counts by 47% in *Urochloa brizantha* (Terra et al. 2024). However, it is necessary to overcome key challenges to successfully implement SynComs, including ensuring environmental dependence, managing prioritisation effects, and leveraging microbe‐microbe signals to coordinate community behaviour in complex field environments.

“Microbiome‐responsiveness” should be considered a key trait in “architecture smart crops,” serving as the central principle of hologenome breeding. Hologenome breeding explicitly selects for genotypes that produce specific exudates to recruit beneficial microbes, thereby maximising phenotypic plasticity. This approach involves selecting for plant genetic loci (QTLs) that govern the recruitment of specific microbial partners, thereby enabling the plant to leverage its “second genome” for adaptive growth. Emerging evidence confirms that plant genotypes exhibit significant variation in this ability. For example, specific *OsCERK1* alleles in rice enhance colonisation by mycorrhizal fungi, leading to improved nutrient acquisition and tiller development.

## 6 Outlook

The first Green Revolution, while successfully boosting crop yield through semi‐dwarf varieties and synthetic fertilisers, critically overlooked the role of the plant microbiota, leading to unintended consequences, such as widespread environmental pollution and substantial greenhouse gas emissions (Li et al. 2024). However, our improved understanding of plant‐microbe interactions offers a promising pathway to mitigate these limitations, by strategically manipulating the root microbiota to unlock unprecedented opportunities, including optimised nutrient cycling, enhanced plant resilience to abiotic and biotic stresses, and increased carbon sequestration (Ge and Wang 2025). This holistic approach, which integrates plant genotype, root exudates, and root microbial communities, is indispensable for developing sustainable agricultural systems capable of withstanding future environmental challenges.

Conclusively, these perspectives contribute to our understanding of crop development as a collaborative endeavour intricately orchestrated by the plant host and its microbial partners. By systematically deciphering the molecular mechanisms underpinning microbial modulation of crop architecture, we are not only advancing fundamental microbiology but also laying the foundation for a new era of sustainable agriculture—an era where the sophisticated power of the plant microbiota is harnessed to cultivate architecture‐smart crops, optimise resource use, and ultimately ensure global food security.

## Author Contributions

**Qinqin Chen:** writing – review and editing, visualization. **Yanlai Yao:** writing – review and editing. **Huan Chen:** supervision, writing – review and editing, writing – original draft, funding acquisition. **Baolei Jia:** writing – review and editing, writing – original draft, funding acquisition, supervision.

## Funding

This work was supported by the Key Research and Development Program of the Xinjiang Uygur Autonomous Region, 2025B04034; Natural Science Foundation of Zhejiang, LZ26C010003; Key Scientific Research Plan Project of Hangzhou, 2024SZD1B24; “Pioneer and Leading Goose+X” R&D Program of Zhejiang, 2025C02267, 2025C01097.

## Conflicts of Interest

The authors declare no conflicts of interest.

## Data Availability

The authors have nothing to report.
